# The Early Diagnosis of Lung Cancer: Critical Gaps in the Discovery of Biomarkers

**DOI:** 10.3390/jcm12237244

**Published:** 2023-11-23

**Authors:** Roberto Gasparri, Angela Sabalic, Lorenzo Spaggiari

**Affiliations:** 1 Division of Thoracic Surgery, IEO, European Institute of Oncology IRCCS, 20141 Milan, Italy; angela.sabalic@ieo.it (A.S.); lorenzo.spaggiari@ieo.it (L.S.); 2 Department of Oncology and Hemato-Oncology-DIPO, University of Milan, 20122 Milan, Italy

**Keywords:** early diagnosis, lung cancer, biomarkers, body fluids, research management

## Abstract

Lung cancer remains the leading cause of cancer-related mortality worldwide. The main issue is the absence of a screening test available in clinical practice; the identification of noninvasive biomarkers is thus an urgent clinical necessity. Currently, low-dose computed tomography (LD-CT) demonstrates a 20% reduction in lung cancer mortality. However, it is not particularly suitable for clinical practice because of its costs, radiation, and false-positive rate. Several studies have therefore focused on research into biomarkers in body fluids. Despite the power of certain molecules to distinguish lung cancer patients from healthy subjects, no biomarker has yet been shown to significantly and reliably influence clinical decisions or to be translated from the laboratory to clinical practice. In this paper, we provide an overview of the peer-reviewed biomedical literature published in the last 10 years on the research regarding biomarkers for the early diagnosis of lung cancer via a comprehensive analysis of the reviews published this past year. Our main objective is to highlight the limitations and strengths of studies on predictive lung cancer biomarkers to stimulate further investigation for early diagnosis. Finally, we discuss future perspectives on managing clinical trials for biomarker research and their integration into clinical practice.

## 1. Introduction

Lung cancer is an aggressive neoplasm and is the leading cause of cancer-related deaths worldwide, with an estimated 1.8 million deaths [1]. The five-year survival rate is associated with the stage of the disease—67% for stage I and 23% for stage III—and the mortality is also strongly associated with late diagnosis [2]. This scenario is aggravated by the absence of a noninvasive screening test, for example, mammography and the fecal occult blood test currently in use for other aggressive neoplasms such as breast cancer and colorectal cancer (survival rate 60–80% respectively). Although low-dose computed tomography (LDCT) has shown a 20% reduction in mortality [3], its application remains limited to the high-risk population (heavy smokers aged 50–80 years), excluding the growing number of young individuals (<50 years) diagnosed with advanced-stage lung cancer [4,5]. Furthermore, the prevalence of false positives leading to unnecessary invasive diagnostic procedures, coupled with the high costs of the methodology, renders it unsuitable for integration into screening initiatives in low-income developing countries [6]. Concerning clinical practice, there is a pressing need for an alternative solution to address the key questions such as noninvasiveness and test reliability while favoring easily obtainable biological samples that can be analyzed with cost-effective tools and reagents, thus making it feasible for adoption even in less industrialized countries. According to the National Institute of Health (NIH), a biomarker is defined as “a characteristic used to measure and evaluate objectively normal biological processes, pathogenic processes, or pharmacological responses to a therapeutic intervention” [7]. In this regard, during the last decade, a considerable number of research studies have focused on the investigation of new technologies for the identification of biomarkers that should be suitable for mass screening, tackling the complexity of the biological and histological heterogeneity of lung cancer. Several biological molecules such as proteins, microRNAs (miRNAs), circulants tumor cells (CTCs), tumor DNA (ctDNA), and volatile organic compounds (VOCs) have been investigated to understand their predictive value. Another key point of early detection is the issue of sample choice. Body fluids such as blood (serum and plasma), urine, stools, exhaled breath, sputum, and saliva meet clinical needs because of their simplicity of collection and noninvasiveness [8,9].

Finally, this review aims to contribute to the literature concerning biomarkers for the early diagnosis of lung cancer. We focused on the state of the art as well as promising biomarkers while discussing the challenges and tips for the discovery of biomarkers and their transition into clinical practice.

### Phases for the Discovery of Lung Cancer Biomarkers

**Figure 1:**
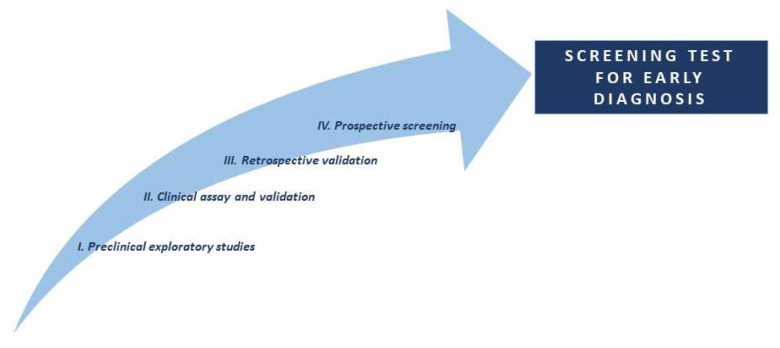
**Diagram of the discovery biomarkers stages: I** comparison of tumor tissue with nontumor tissue to identify characteristics unique to the neoplasm, to identify possible biomarkers (e.g., proteins, mRNA, genes). **II.** Designing a case–control study to assess whether the biomarker is uniquely expressed in both tumor tissue and other noninvasive biological samples (e.g., blood) with the aim of distinguishing cancer patients from healthy subjects. **III.** Collection of noninvasive samples from cancer patients who have not yet received a diagnosis and from subjects who have not developed cancer. This aspect could confirm the biomarker’s ability to detect cancer in a preclinical stage, making it suitable as a screening test. **IV.** Individuals who tested positive with biomarkers are referred for further diagnostic evaluation. This phase also helps to identify the number of false-positive cases undergoing further assessment.

The investigation of biomarkers and their translation into clinical practice are the main issues in the development of a screening test. In 2001, Pepe et al. proposed four consecutive phases to reach the final validation of the biomarkers (Figure 1).

The first phase is the identification of the specific biomarkers of the neoplasia by comparing the analysis of tumor and healthy tissue. A crucial factor in the first phase is the stratification of the study cohort, particularly the control group, to ensure that other factors such as age, gender, race, and possibly lifestyle-related characteristics (smoking habits) do not influence the biomarker’s expression, which would result in an overlap between cases and controls. In the second phase, the biomarkers detected in the first phase are searched in samples that do not require invasive procedures for collection (e.g., blood, urine, and respiratory exhalation). In phase III, a comparison is made between individuals who have cancer (not diagnosed at the time of biomarker analysis) and individuals who have not developed the disease. In phase IV, patients who test positive in the screening (using only the biomarker) are referred for further diagnostic evaluation. This phase also helps to identify the number of false-positive cases undergoing further assessment.

Although the research path for a biomarker may seem simple, in practice, it is a highly complex and expensive process. The validation phases require a large number of samples to ensure the appropriate statistical validity of the data, as well as samples that reflect the biological variability of the population [10].

## 2. Materials and Methods

### Selection of Articles

**Figure 2:**
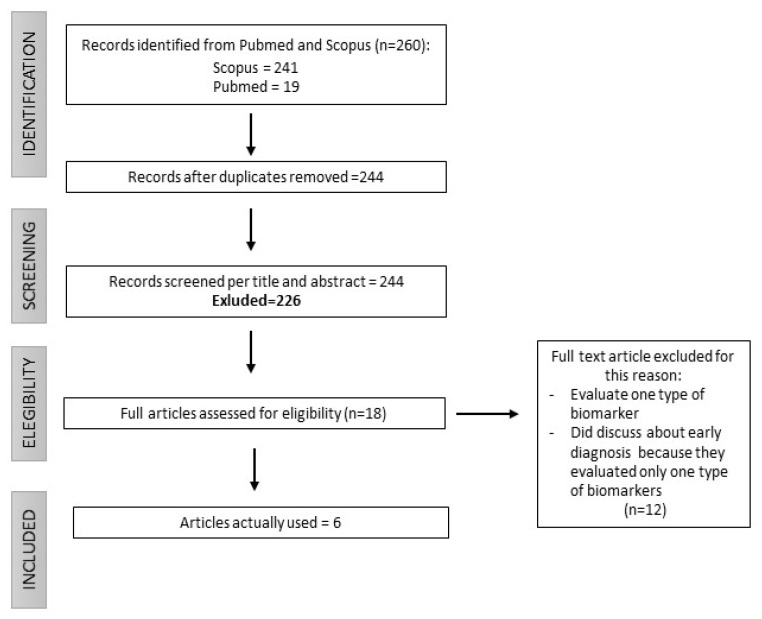
**Flowchart**.

We studied the review publications related to biomarkers for the early diagnosis of lung cancer that were indexed on the PubMed and Scopus scientific literature databases, using the keywords *“lung cancer”* AND *“early diagnosis”* AND *“biomarkers”*. Specifically, we focused on reviews published between 2022 and 2023 that encompassed the literature from the past 10 years. The search produced 260 results; after the removal of duplicates, the review articles were screened by examining the titles and abstracts. After a further screening based on the reading of the entire article, six reviews were selected for the final text (Figure 2).

Through the comparative analysis of the reviews, we assessed the biomarkers investigated to date, the types of samples used, and the analytical techniques employed. The ultimate goal was to identify which studies are currently in the validation phase and which biomarkers hold future potential as predictive elements for lung cancer. Additionally, we aimed to shed light on the limitations and key factors that lead to a biomarker reaching the validation phase.

## 3. Results: Current and Promising Lung Cancer Biomarkers

### 3.1. Circulating Blood Proteins and Autoantibodies

Circulating proteins can stem from various sources, including the overexpression of cancer cells, increased secretion from diseased tissue, or inflammation linked to malignancy. The proteome has been widely studied in the oncological field to identify serum proteins as potential biomarkers for the early diagnosis of lung cancer. Among the most interesting studies conducted in the last 10 years, CancerSEEK reported a panel of eight proteins (CA-125, CEA, HGF, Myeloperoxidase, OPN, Prolactin, and TIMP-1) effective in distinguishing lung cancer patients from healthy controls [11]. Moreover, the combination with cfDNA increases the sensitivity of this protein panel [11,12]. In another study, a panel of three proteins and one autoantibody (NY-ESO-1) were assessed, and a sensitivity of 71% and specificity of 88% were observed [13]. Mazzone et al. performed a separate clinical trial with the same test (PAULA), demonstrating a sensitivity and specificity of 49% and 96%, respectively [14]. A prospective proteomic study based on two proteins (LG3BP and C163A) integrated with clinical and imaging features showed a sensitivity of 97% and a specificity of 44% [15]. A more recent project involves the development of a 36-protein multiplex assay for the risk assessment of lung cancer. However, more studies should be conducted to demonstrate that these approaches are suitable to implement in clinical practice [9]. Cancer cells stimulate the immune system through the release of protein inducing the production of circulating autoantibodies against tumor-associated antigens (TAAs). EarlyCDT, a panel of seven autoantibodies (p53, NY-ESO-1, CAGE, GBU4-5, HuD, MAGEA4, and SOX2), is commercially available as a blood test to assess the risk of malignancy in people with solid pulmonary nodules [16]. A clinical trial with EarlyCDT on a symptomatic lung cancer patient showed a sensitivity of 41% and a specificity of 91%, and a follow-up study on a high-risk cohort revealed a sensitivity and a specificity of 37% and 91%, respectively [17]. Moreover, Qiang Du et al. tested p53, PGP9.5, SOX2, GAGE7, GBU4-5, MAGEA1, and CAGE and found no statistically significant difference between stages I/II and III/IV, concluding that the test is capable of detecting both early and advanced stages. This phenomenon could be related to the amplification of the immune system. Further studies will be needed to understand the potential prognostic power of proteins and TAAs [18,19]. Their stability in the serum allows them to be detected via immunoenzymatic assays (ELISAs) and makes TAAs possible biomarkers for the early diagnosis of lung cancer [20].

### 3.2. microRNA (miRNAs)

MiRNAs are small noncoding RNAs that are involved as regulators of gene expression at the post-transcriptional level. They can be aberrantly expressed in many pathological processes as well as in cancer. MiRNAs can be detected in different body fluids such as urine, sputum, and blood (serum and plasma) [21]. In 2002, Calin et al. reported the involvement of microRNAs in lung cancer pathogenesis [22]. They preserve their stability from initial development to metastasis formation, making them appealing biomarkers for the diagnosis and prognosis of lung cancer [8,9,21]. An early study conducted on lung tissue detected 12 miRNAs expressed differently between lung cancer tissue and benign lung tissue [23]. In addition, studies on miRNAs in sputum have shown that the combination of multiple miRNAs can differentiate lung cancer patients from healthy individuals with a sensitivity of 73% to 80% and a specificity of 91% to 96% [24]. Two further studies have compared different miRNA panels in lung cancer patients before and after lung cancer resection and in healthy controls. Le HB et al. showed an increased expression in the serum of miR-21, miR-205, miR-30d, and miR-24 before lung cancer surgery. The same miRNA was upregulated in the serum of early-stage lung cancer patients in comparison to healthy subjects, suggesting their role as a screening biomarker as well as for postoperative disease relapse [25]. Moreover, an 18-month postsurgery follow-up conducted by Leidinger et al. demonstrated a significant reduction in the expression levels of miRNA over time after the surgery [26]. Currently, the miR-Test [27] and MSC (microRNA signature classifier) [28] are undergoing validation. The serum signature of miRNA identified in high-risk subjects enrolling in a screening program with LDCT showed a sensitivity and specificity of 77.8% and 74.8%, respectively [27]. Sozzi et.al. based on 24 miRNA expression ratios stratified the population into low, inter-mediate, or high risk of lung cancer [29]. Their study revealed 87% sensitivity and 81% specificity. Both studies exhibited a reduction in the LDCT false-positive rate [27,28,29].

### 3.3. Circulating Tumor Cells (CTCs) and Circulating Tumor DNA (ctDNA)

CTCs are derived from the primary tumor mass. During this process, the cells detached from the tumor mass enter the circulatory stream. CTCs were evaluated in a group of 168 patients with chronic obstructive pulmonary disease (COPD) followed with annual CT scans for 4 years. It was found that COPD patients who tested positive for CTCs in the annual CT screening developed lung nodules 1–4 years later. These studies suggest that CTCs could be used for early diagnosis [30,31]. Another study showed that the sensitivity and specificity of CTCs for diagnosing lung cancer were 73.2% and 84.1%, respectively [32], while Wang et al. obtained a sensitivity of 77.7% and a specificity of 89.5%. The comparison between the sensitivity of stage I and stage II revealed that the two values almost overlapped (69.8% and 72.2%) [33]. A study of a larger lung cancer patient cohort demonstrated sensitivity and specificity values similar to other studies, but with the combination of CEA and additional biomolecules, these values could be increased to 84.21% and 88.78%, respectively [32]. Emerging research with negative enrichment fluorescence in situ hybridization methods or the FISH approach demonstrated that the sensitivity and specificity were increased (89–100%) [31,33,34].

ctDNA is a part of cell-free DNA derived from tumor cells. The concentration of ctDNA in plasma varies from 0.01% to 90% [35]. Newman et al. observed a 100% rate of ctDNA in patients with stage II-IV lung cancer, while a 50% rate was observed in early-stage patients [36]. The combination with protein showed a specificity of 99% and a sensitivity of 59% [11,12]. Using deep sequencing (CAPP-seq), Chabon et al. investigated cancer profiling to analyze the ctDNA. This approach demonstrated that ctDNA levels were low in early-stage lung cancer. The same research group developed and validated a machine learning method (Lung-CLiP) using the findings described above in conjunction with other molecular features, and a specificity of 96% was achieved [37]. Phomaryova demonstrated that in lung cancer patients, the concentration of ctDNA is eight times higher than that in healthy individuals [38]. Furthermore, studies report that high concentrations of circulating ctDNA are correlated with a worse clinical outcome [34]. However, ctDNA has demonstrated poor sensitivity, and most patients have levels of less than 0.1%, which is challenging to detect in the blood [9].

### 3.4. Future Directions and Challenges: Volatile Organic Compounds (VOCs)

Since the 1970s, volatile organic compounds have been used in the field of medicine [39]. Lung cancer studies emphasize the presence of VOCs in exhaled breath [40]. The most widely used approach for the analysis of respiratory VOCs is gas chromatography combined with mass spectrometry (GC/MS) [41]. This method has shown a discriminatory power to detect the specific volatile compounds of lung cancer patients. In one study, GC/MS combined with artificial neural networks showed a sensitivity of 80% and specificity of 91% [42]. In a prospective pilot study, Peled et al. demonstrated the potential of breath analysis to distinguish malignant nodules from benign nodules in high-risk subjects [43]. Another promising measurement device in the field of early diagnosis is the electronic nose (e-nose). This emergent technology is based on the binding of VOCs to different sensors or sensor arrays within handheld devices. The investigators analyzed 214 breath samples using an e-nose with 11 gas sensors. The experimental results revealed an accuracy of 95.75%, a sensitivity of 94.78%, and a specificity of 96.96% [44]. Shlomi D et al. compared patients with benign lung nodules and patients with lung cancer. Moreover, the lung cancer group was divided into two subgroups: patients who harbored the EGFR mutation and lung cancer patients with wild-type EGFR. This study showed the discriminatory power to distinguish the early LC from benign nodules and had 87% accuracy [45]. Two other studies used an e-nose to detect a specific lung cancer signature (in lung cancer patients vs. high-risk healthy controls) with a sensitivity of 81% and specificity of 91% [46]. Moreover, Gasparri et al. demonstrated that an e-nose with 12 sensors has a greater sensitivity to lung cancer at stage I with respect to stage II/III/IV (92% and 58%, respectively) [47]. Additionally, a recent multicentric case–control study yielded a sensitivity of 95% and a specificity of 49% [48].

So far, more than 100 volatile urinary biomarkers have been suggested as being related to cancer. Urinary VOC patterns in cancer patients are often different from those found in the urine samples of control subjects, and these differences also depend on cancer type and stage [49]. In 2023, investigators isolated for the first time five specific VOCs of early-stage lung cancer (I/II) with a specificity and sensitivity of 85% and 90%, respectively [50]. Results with greater robustness are warranted before these may be fully integrated into workflows or incorporated into clinical guidelines.

All suitable biomarkers are shown in Table 1.

## 4. Discussion

The early diagnosis of lung cancer ranks among the most crucial health issues. The five-year survival is strongly correlated with stage (90% stage I vs. 10% stage IV) [51]. Considerable advances have been made in metastatic lung cancer diagnosis and treatment by finding numerous disease subtypes defined by specific oncogenic driver mutations (EGFR, ALK, ROS1, BRAF, HER2, MET, RET or KRASG12C, and PD-L1). This has led to the development of a range of molecularly targeted therapies, which have exerted a significant impact on patient survival rates [52]. By contrast, although numerous studies have been conducted to search for useful biomarkers for early diagnosis, none of the investigated molecules have been incorporated into clinical practice. Currently, in a clinical setting, serum tumor markers are increasingly being used as a supplement to radiological examinations (CT and PET) for therapy monitoring and disease recurrence. Studies on lung cancer patients have demonstrated that proteins such as CYFRA 21-1, CEA, and NSE can be used to determine the lung cancer subtype or are correlated with the stage and prognosis of the disease. Additionally, the integration of ctDNA into the protein panel has shown potential for therapy monitoring [11,13,14,15]. However, the serum concentration of these biomarkers also increases in the presence of other malignancies, rendering them nonspecific. Thus, despite the emerging evidence of their potential in early diagnosis and treatment monitoring, they have not yet been incorporated into clinical guidelines. Further studies will be necessary to determine whether proteomic analyses could be cost-effective for lung cancer screening, even in low-income countries [9]. On the other hand, TAAs can be detected in the blood by means of the ELISA assay, one of the most specific and straightforward tests for detecting circulating biomolecules, widely used in research settings and clinics worldwide [53]. Furthermore, one study reported that lung cancer patients have TAA levels that are 30% higher than those in healthy individuals [54]. Du Q et al. revealed that TAA levels do not differ among different stages of the disease, and they can be used to detect both early and advanced stages of lung cancer, suggesting that this phenomenon may be related to immune amplification. This characteristic could be crucial for early diagnosis [18]. However, studies described in the peer-reviewed biomedical literature have shown a low test sensitivity (30–40%), limiting their use in detecting high-risk subjects [16,17,18,19]. Therefore, additional studies will be needed to understand the combination of antibodies with greater sensitivity and specificity for early-stage lung cancer.

Concerning microRNAs, a significant number of research studies have explored their potential role as a biomarker in the early diagnosis of lung cancer. MicroRNA has been studied alone or in combination with other biomolecules, showing high sensitivity and specificity in distinguishing lung cancer subjects from healthy controls [24,26,29]. As described in the Results section, the miR-Test and MSC are the only studies currently ongoing and are pending validation. Moreover, the miR-Test has presented accuracy, sensitivity, and specificity of less than 80%, as well as detection sensitivity between stage I (69%) and stages II/III (71%), which is not particularly significant [27]. At the same time, the MSC has shown a greater effect only in combination with CT [29]. All these miRNA studies conducted so far suffer some degree of limitation such as a small sample size, the selection and inclusion of patients in advanced stages, epidemiological diversity, and poor homogeneity in experimental protocols [8,19].

Other interesting biomolecules are the CTCs. These are already used in thoracic oncology for the molecular characterization of lung cancer, suggesting their potential role in the early diagnosis of lung cancer. Indeed, recent data have shown that lung cancer patients have CTC levels ten times higher than patients with other types of cancer, and their presence in the circulation is associated with a worse prognosis [35]. However, CTC isolation and detection remain complex procedures that require the development of new technologies. Furthermore, high specificity (96–100%) and low sensitivity (26–68%) have been reported in the literature [32,33]. ctDNA, like CTCs, has a circulating concentration ranging from 0.01% to 90% depending on the lung cancer burden and its progression [35]. As demonstrated in the literature, its concentration is much higher in advanced stages than in early stages (100% vs. 50%), resulting in levels eight times higher than in healthy individuals [11,12,38]. While these characteristics make ctDNA a promising biomarker for early diagnosis, its low plasma quantity and half-life have challenged its clinical applicability. Further research on biological samples other than plasma could be useful in assessing the future applicability of ctDNA. However, future studies will be necessary to explore the diagnostic power of CTCs and ctDNA for early lung cancer diagnosis and their utility in clinical practice.

During the last decade, the volatolomic profile has garnered much interest in the field of the early diagnosis of lung cancer. Various types of VOCs can be detected in exhaled breath because they have low solubility in the blood and are thus excreted in the breath within minutes of their formation. Several analytical methods for VOC detection have been recently developed. The most widely used analytical method is gas chromatography–mass spectrometry (GC/MS). However, MS-based analysis is expensive and requires skilled staff. Other innovative and cost-effective tools are available for potential future applications in clinical practice, such as artificial sensors (electronic nose). Studies have demonstrated the ability of the electronic nose (e-nose) to distinguish between lung cancer patients and healthy subjects, particularly with greater sensitivity in diagnosing stage I lung cancer compared to stages II, III, or IV. Furthermore, the combined use of GC/MS and e-nose could enhance the method’s specificity, sensitivity, and accuracy. Recent research has unveiled the potential of urinary volatile compounds for early nonsmall cell lung cancer (NSCLC) diagnosis, indicating the ability of GC/MS to detect not only specific volatile compounds related to the lung cancer group but also to discriminate specific compounds associated with the early stages. Future research will be necessary to implement the results obtained thus far.

## 5. Study Limitations

Literature studies underline the potential of several biomarkers such as circulating blood proteins and autoantibodies, miRNA, CTCs, ctDNA, and VOCs to differentiate lung cancer patients from healthy subjects, through noninvasive body fluid analysis. However, some limitations such as a small sample size, limited stratification of the population including few early stages of lung cancer patients [31], the lack of a long follow-up, and the lack of a standard approach limit scientific robustness and their translation in clinical practice.

## 6. Future Perspectives

The near future should be focused on establishing a multicenter clinical trial involving a large number of participants, with the aim of improving the population stratification involved in the study. The group of lung cancer patients should be stratified based on the radiological characteristics of the nodule (e.g., ground-glass opacity, solid nodule, and spiculated nodule) alongside histological factors (TNM staging), with a particular emphasis on early stages. Simultaneously, the group of high-risk healthy subjects should be stratified based on their family cancer history, smoking history, and existing lung conditions. Furthermore, studies should include both prospective and retrospective analyses, along with the planning of a follow-up to monitor the behavior of biomarkers in high-risk healthy subjects, in order to identify the potential presence of a malignant lung nodule in individuals who have not yet received a diagnosis.

The results should be analyzed by establishing a universal database that is accessible to a global network of research centers dedicated to early lung cancer diagnosis. Data sharing is paramount for resource synergy and optimization, reducing costs in biomarker research. Ideally, a consortium would be established, whereby biomarkers for mass population screening are discussed and evaluated. Each participant should use a variety of approaches and data collection methods and make them transparently available in the public domain. Furthermore, great technological strides have witnessed the development and deployment of artificial intelligence and machine learning tools that can be used to process, overlay, and integrate molecular biomarkers with clinical and epidemiological data.

**Figure 3:**
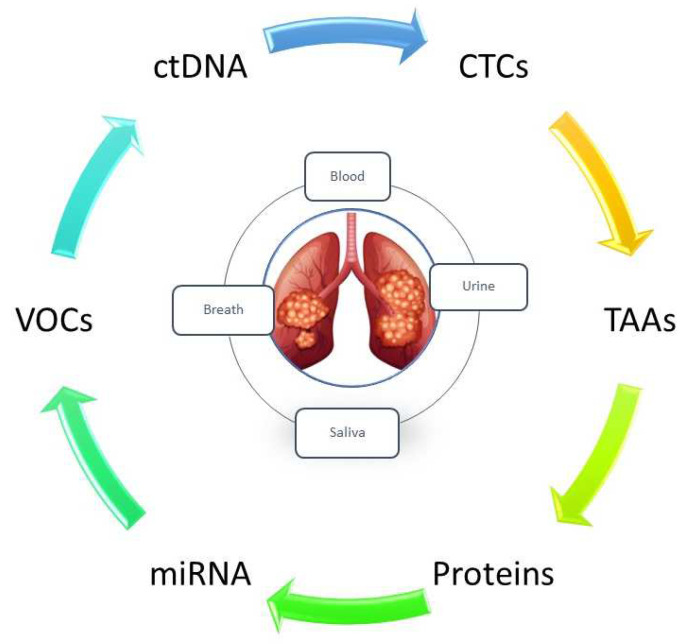
Overview of liquid biopsy and promising biomarkers for the early diagnosis of lung cancer.

The milestone we aspire to reach is a noninvasive and relatively cost-effective diagnostic tool that can significantly impact clinical decision making. This tool should be robust and readily implementable in the clinical practice of all healthcare facilities, even those in resource-constrained countries (Figure 3).

## 7. Conclusions

In conclusion, despite the potential role demonstrated by the described biomarkers in distinguishing patients with lung cancer from healthy individuals, to date, there are no biomarkers available in clinical practice for the early diagnosis of lung cancer. Future research should focus more on population stratification and the use of standardizable and reproducible methodologies, as well as on the organization of long-term follow-ups, particularly for the population at high risk. Collaboration among multiple researchers could play a fundamental role.

## Figures and Tables

**Table 1:** **Selected lung cancer biomarkers**.

Study	Population	Method	Biomarkers	Main Results
Xu BJ [11]	40 LC 8 HR	MALDI-MS	Proteins	75% accuracy
Doseeva V [13]	75 LC 75 HR	IMMUNOASSAY xMAP	Proteins and autoantibody	77% sensitivity 80% specificity
Mazzone PJ [14]	155 LC 245 HR	IMMUNOASSAY MAGPIX	Proteins and autoantibody	74% sensitivity 80% specificity
Silvestri GA [15]	29 LC 149 HR	MS	Proteins	97% sensitivity 44% specificity
Chapman CJ [17]	235 LC 266 HR	ELISA	Autoantibodies	92 % accuracy
Du Q [18]	305 LC 74 HR	ELISA	Autoantibodies	56.53% sensitivity 91.60% specificity
Yu L [24]	64 LC 58 HR	qRT-PCR	miRNA	80.6% sensitivity 91.7% specificity
Montani F [27]	74 LC 115 HR	NA	miRNA	77.8% sensitivity 74.8% specificity
Sozzi G [29]	69 LC 870 HR	PCR	miRNA	87% sensitivity 81% specificity
Yu Y [32]	153 LC 93 H	RT-PCR + FISH	CTCs	67.2% sensitivity for stage I 84.1% specificity
Katz RL [33]	107 LC 100 H	FISH	CTCs	89% sensitivity 100% specificity
Newman AM [36]	13LC 13 H	CAPP-Seq	ctDNA	96% specificity
Ponomaryova AA [38]	60 LC 32 H	TaqMan PCR (MSP)	cirDNA	87% sensitivity 75% specificity
Rudnicka J [42]	86 LC 41 H	GC/MS	VOCs	80% sensitivity 91.23% specificity
Shlomi D [45]	89 LC 30 H	eNOSE	VOCS	83% accuracy 79% sensitivity 85% specificity
McWilliams A [46]	25 LC 166 H	eNOSE	VOCs	80% accuracy
Gasparri R [47]	70 LC 76 H	eNOSE	VOCs	81% sensitivity 91% specificity
Hanai Y [49]	20 LC 20 H	GC/MS	VOCs	95% sensitivity 70–100% specificity
Gasparri R [50]	46 LC 81 H	GC/MS	VOCs	85% sensitivity 90% specificity

LC = lung cancer patients; H = healthy subjects.
